# Centenarians—the way to healthy vascular ageing and longevity: a review from VascAgeNet

**DOI:** 10.1007/s11357-024-01467-8

**Published:** 2024-12-27

**Authors:** Sabrina Summer, Maria Borrell-Pages, Rosa-Maria Bruno, Rachel E. Climie, Konstantina Dipla, Aysenur Dogan, Kseniia Eruslanova, Emil Fraenkel, Francesco Mattace-Raso, Christopher J. A. Pugh, Keith D. Rochfort, Mark Ross, Lynn Roth, Arno Schmidt-Trucksäss, Dennis Schwarz, James Shadiow, Yahya Sohrabi, Jannik Sonnenberg, Olga Tura-Ceide, Bilge Guvenc Tuna, Josep Julve, Soner Dogan

**Affiliations:** 1 Department for Biomedical Research, University for Continuing Education Krems, Krems, Austria; 2 Molecular Pathology and Therapeutic of Ischemic and Atherothrombotic Diseases, Institute de Recerca Sant Pau (IR-Sant Pau), Barcelona Spain. CIBERCV, Instituto de Salud Carlos III, Madrid, Spain; 3 Menzies Institute for Medical Research, University of Tasmania, Hobart, Australia; 4 Department of Sports Sciences at Serres, Aristotle University of Thessaloniki, Thessaloniki, Greece; 5 Department of Medical Biology, School of Medicine, Yeditepe University, Istanbul, Türkiye; 6 Russian Gerontology Research and Clinical Centre, Pirogov Russian National Research Medical University, Moscow, Russia; 7 Department of Internal Medicine, University of Košice, Košice, Slovakia; 8 Department of Internal Medicine, Erasmus University Rotterdam, Rotterdam, Netherlands; 9 School of Sport & Health Sciences, Cardiff Metropolitan University, Cardiff, UK; 10 School of Nursing, Psychotherapy and Community Health, Dublin City University, Dublin, Ireland; 11 Institute of Life and Earth Sciences, Heriot-Watt University, Edinburgh, UK; 12 Laboratory of Physiopharmacology, University of Antwerp, Antwerp, Belgium; 13 Department of Sport, Exercise and Health, Division of Sport and Exercise Medicine, University of Basel, Basel, Switzerland; 14 Department of Cardiology I–Coronary and Peripheral Vascular Disease, University of Münster, Münster, Germany; 15 School of Kinesiology, University of Michigan, Ann Arbor, MI USA; 16 Department of Medical Genetics, Third Faculty of Medicine, Charles University, Prague, Czechia; 17 Institut d’Investigació Biomèdica de Girona, Girona, Spain; 18 Department of Biophysics, School of Medicine, Yeditepe University, Istanbul, Türkiye; 19 Endocrinology, Diabetes and Nutrition Group, Institut de Recerca SANT PAU, Barcelona, Spain; 20 CIBER de Diabetes y Enfermedades Metabólicas Asociadas (CIBERDEM), Instituto de Salud Carlos III, Madrid, Spain; 21 Université Paris Cité, INSERM, Paris Cardiovascular Research Center-PARCC, Paris, France; 22 Clinical Pharmacology Unit, AP-HP, Hôpital Européen Georges Pompidou, Paris, France

**Keywords:** Healthy vascular ageing, Centenarians, Cardiovascular diseases, Health span

## Abstract

The prevalence of centenarians, people who lived 100 years and longer, is steadily growing in the last decades. This exceptional longevity is based on multifaceted processes influenced by a combination of intrinsic and extrinsic factors such as sex, (epi-)genetic factors, gut microbiota, cellular metabolism, exposure to oxidative stress, immune status, cardiovascular risk factors, environmental factors, and lifestyle behavior. Epidemiologically, the incidence rate of cardiovascular diseases is reduced in healthy centenarians along with late onset of age-related diseases compared with the general aged population. Understanding the mechanisms that affect vascular ageing in centenarians and the underlying factors could offer valuable insights for developing strategies to improve overall healthy life span in the elderly. This review discusses these key factors influencing vascular ageing and how their modulation could foster healthy longevity.

## Introduction

Alongside the rise in average life expectancy and reduced fertility, the number of people showing successful ageing is steadily increasing. Successful ageing is defined here as high physical, psychological, and social functioning during ageing without the development of major diseases. People with an extended life expectancy could be classified into near-centenarians (95–99 years), centenarians (> 100 years), semi-supercentenarians (> 105 years), and super-centenarians (> 110 years) [1, 2]. All these individuals reached the extreme decades of human life and have decreased prevalence or delayed incidence of chronic complications related to age-associated diseases [2–5]. Based on this and for the sake of simplicity, near-centenarians, centenarians, semi-supercentenarians, and super-centenarians are, unless specifically stated, summarized in this article under the term “centenarians.”

Studies on centenarians offer valuable insights into longevity, healthy ageing, disease resistance, and the broader societal implications of an ageing population. In the context of cardiovascular diseases (CVD), centenarians are an excellent paradigm, whereby the incidence of macro- and microvascular cardiac complications is lower than that of the general aged population. Therefore, profound understanding of the dynamics of the vasculature in centenarians is particularly intriguing as it may shed light on their remarkable resilience to age-related vascular changes. Despite the natural wear and tear on blood vessels over time, centenarians often exhibit preserved vascular function and structure [6], suggesting that the ageing process of the vasculature in large and small blood vessels might be delayed in these individuals.

The development of the vascular system takes place during embryogenesis when elastin, the main responsible factor for arterial wall elasticity, is synthesized [7, 8]. Ageing induces a progressive loss of tissue and organ function. It progressively deteriorates blood vessels and is considered a prominent risk factor for CVD. In this context, the microvasculature is essential for the physiology of tissues and organs. Conceivably, age-induced vessel alterations, such as loss of elasticity, wall thickening, and increased stiffness [9, 10], may negatively influence their functionality.

In the clinical setting, vascular ageing is commonly defined as a metabolic process characterized by progressive age-related alterations that affect both mechanical and structural properties of the vascular wall that will lead to structural and functional changes including epigenetic modification, cellular senescence, oxidative stress related to mitochondrial damage, endothelial dysfunction—a decline in the normal functioning of the inner lining of blood vessels, and increased arterial stiffness [11].

Identifying the key factors linked to longer life spans and understanding their physiological and molecular mechanisms might pave the way to providing valuable insights that could be applied globally to improve human health. Recent survey reports that Japan has the highest absolute number of centenarians per 10.000 people, followed by the USA [12]. Despite their cultural and societal differences, these two countries share an advanced medical care and increased public health measures. In terms of diet, however, they are quite diverse. While the Japanese diet is mostly plant-based associated with lower blood pressure and cholesterol levels, reducing the risk of heart disease, the American diet, despite the growing focus on health-conscious eating, is characterized by high intakes of processed foods and red meat with a higher risk of heart diseases. While they differ in lifestyle and culture, there seems to be common factors that contributed to the increased number of centenarians in these countries.

**Fig. 1:**
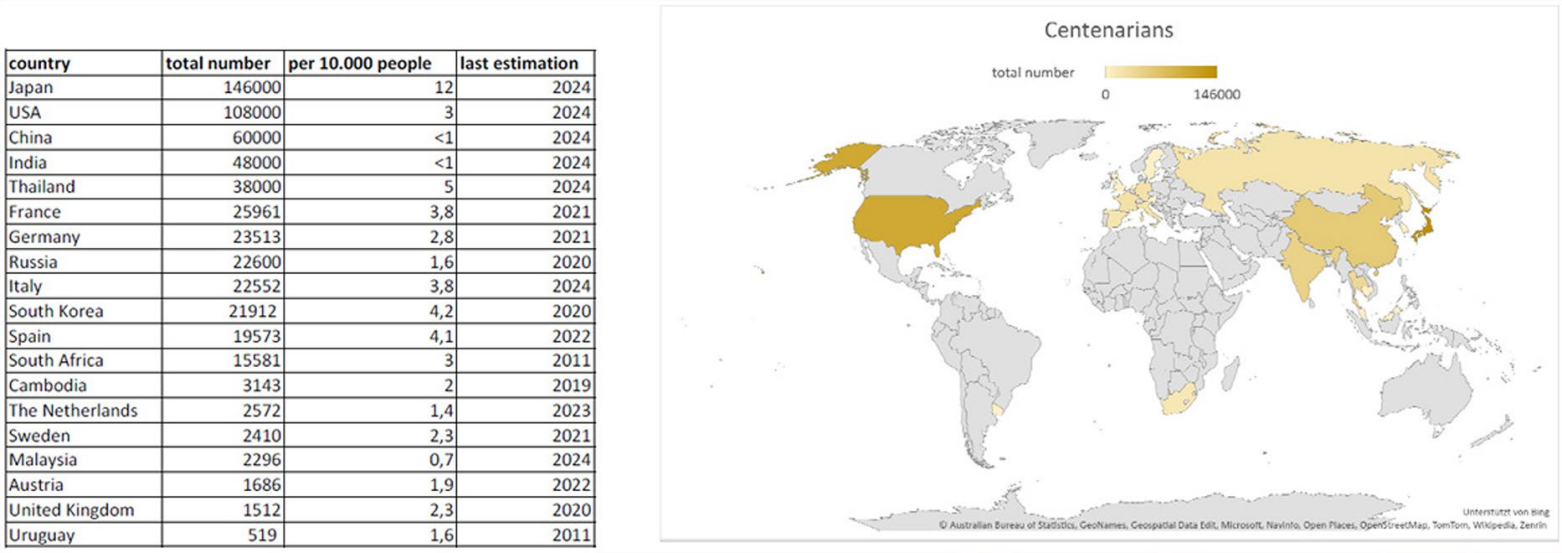
Estimated total number of centenarians in selected countries around the world. Japan and the USA are the top two countries with the highest number of centenarians worldwide [12]. The map illustrates the selected countries in brown tones depending on the number of centenarians in the corresponding countries (dark brown—high number, biscuit—low number, and white—not indicated)

The purpose of the present review is to highlight both intrinsic and extrinsic factors closely linked to healthy ageing in countries with longer life expectancies, with a specific focus on their role in reducing CVDs (Fig. 1). However, we will not deepen into the description of cellular mechanisms of vascular ageing, as they were addressed in detail previously [13]. In this review, we will assess how these factors are differentially influenced in centenarians and protect them against CVD, which has not been comprehensively discussed so far.

## The vasculature of centenarians

While centenarians experience age-related changes in the vasculature, such as arterial stiffening and endothelial dysfunction, to some extent, their vascular systems often remain healthier compared to the average elderly population [14, 15]. This may be associated to a combination of genetic factors, adaptive immune responses, and healthier lifestyles, which help mitigate the damage caused by inflammatory and oxidative processes over time.

Although ageing is considered a major risk factor for CVDs [16], the proportion of centenarians accumulating cardiovascular risk factors is relatively low compared with the general aged population [17, 18]. Compelling evidence suggests that certain intrinsic characteristics of centenarians’ vasculature could provide protection against vascular dysfunction. In this context, studies on vascular ageing in centenarians investigate whether their blood vessels are better adapted or more resistant to typical age-related changes, potentially contributing to their ability to reach an advanced age while preserving cardiovascular health. One key feature in centenarians is a better balance between pro-inflammatory and anti-inflammatory mechanisms within the vascular system [19, 20]. Unlike most older adults who experience heightened levels of chronic inflammation, centenarians often maintain lower levels of inflammatory markers such as interleukin (IL)−6 and C-reactive protein (CRP) [21], associated with vascular damage. Additionally, they tend to preserve better endothelial function, which allows their blood vessels to retain the ability to dilate and regulate blood flow [14, 22]. This preservation of endothelial health, along with their lower rates of hypertension and arterial plaque formation [23] (Table 1), suggests that centenarians may have a network of protective mechanisms that delay or reduce the severity of vascular ageing.

**Table 1:** Vascular ageing measurements related to cardiovascular risk in centenarians

Characteristics of subjects	Indicators	Study design	Follow-up period (years)	Main observations	reference
*Imaging*					
697 adults (26–85 years), vascular ageing indicators measured at least twice between 2007 and 2018	Systolic blood pressure, ankle-brachial index, heart rate, common carotid diastolic diameter, carotid artery mean blood velocity, blood flow, carotid intima-media thickness, carotid-femoral pulse wave velocity, and vascular ageing index	Longitudinal; repeated-measures analysis	6.7	Carotid IMT had the strongest relationship with chronological age in the longitudinal analysis in both sexes	[34]
*Biochemical biomarkers*					
319 healthy subjects (154 men, 165 women; 21–105 years), no history of hypertension, diabetes mellitus, or atherosclerotic disease	Arterial wall thickness (IMT); the presence of plaques assessed by B-mode ultrasound	Cross-sectional	N/A	Increased carotid IMT; plaque prevalence decreased in centenarians	[23]
Healthy 146 volunteers (21–96 years); predominantly sedentary population and in endurance trained older men relative to their less active age peers	Carotid arterial pressure pulse augmentation index (AGI), using applanation tonometry, and aortic pulse wave velocity (APWV)	Cross-sectional	N/A	endurance training, indexed by VO_2_max was associated with reduced arterial stiffness in older men	[177]
Individuals turning 100 years old in 1995 (*n* = 106) and 2015 (*n* = 238)	Cardiovascular profile was obtained by measured blood pressure, electrocardiogram (ECG), and information on medication. Hypertension defined as systolic blood pressure (SBP) ≥ 140 mmHg and/or diastolic blood pressure (DBP) ≥ 90 mmHg	Cross-sectional (comparative study of 2 birth cohorts born 20 years apart)	N/A	ECG findings were similar in the two cohorts, no amelioration of hypertension between cohorts; but the most recent born cohort (1915) was treated more intensively with cardiovascular drugs than the earlier (1895)	[178]
Population-based prospective cohort study including 3,315 participants (60–105 years, 64.6% women), examined from 2001–2004 through 2013–2016	The longitudinal trajectories of systolic BP (SBP), diastolic BP (DBP), pulse pressure (PP), and mean arterial pressure (MAP), age estimated using linear mixed-effects models	Longitudinal	9–15	Around 80 years old people with heart disease showed steeper declines in SBP and PP than those without	[179]
*Metabolic/clinical-based data*					
The offspring of 192 centenarian subjects enrolled in the nationwide New England Centenarian Study recruited and enrolled. Controls consisted of offspring from non-long-lived parents	Study participants were sent questionnaire packets that included the following: (a) a demographic and health questionnaire, (b) a functional status questionnaire, and (c) a physical examination form	Cross-sectional	N/A	Centenarian offspring showed 56% reduced relative prevalence of heart disease, 66% reduced relative prevalence of hypertension, and 59% reduced relative prevalence of diabetes after multivariate adjusted analyses	[180]
Offspring of centenarians (265 subjects) showed a different prevalence of MetS in comparison to the offspring of non-long-lived parents (controls, 101 subjects)	Past and current disease history of myocardial infarction, hypertension, hypercholesterolemia, diabetes. MetS was defined according to the Third Report of the National Cholesterol Education Program Expert Panel on Detection, Evaluation, and Treatment of High Blood Cholesterol in Adults (NCEP-ATP III) criteria [181]	Cross-sectional	N/A	Despite having similar prevalence of MetS and all its components in both groups, the prevalence of past cardiovascular events, hypertension, hypercholesterolemia was significantly reduced in the presence of MetS, centenarians’ offspring	[182]
1427 individuals from three longitudinal cohort studies, including 36 supercentenarian (> 110 years), 572 semi-supercentenarians (105–109 years), 288 centenarians (100–104 years), and 531 very old people (85–99 years)	Plasma levels of NT-proBNP, erythropoietin, interleukin-6, TNF-alpha, Angptl2, cystatin C, holinesterase Activity, albumin, g213Gly polymorphism at *SOD3* locus (rs1799895)	Prospective, cross-sectional	N/A	Low NT-proBNP levels are statistically associated with a survival advantage to supercentenarian age. Only low albumin is associated with high mortality across age groups	[18]
Population-based prospective study of 764 participants (50–89 years) from a community in Copenhagen, Denmark, 658 participants provided blood and urinary samples and were examined from 1998 to 2000	NT-proBNP vs CRP and urinary albumin/creatinine ratio	Longitudinal	5	NT-proBNP was revealed as a stronger risk biomarker for cardiovascular disease and death than CRP was in non-hospitalized individuals aged 50 to 89 years	[183]

*CRP* c-reactive protein, *ECG* electrocardiogram, *IMT* intima-media thickness, *MetS* metabolic syndrome, *NT-proBNP* N-amino terminal fragment of the prohormone brain natriuretic peptide

Genetics likely play a substantial role in this vascular resilience, with some centenarians possessing gene variants that protect against excessive inflammation, lipid accumulation, and oxidative damage [24, 25]. Coupled with this, many centenarians maintain active lifestyles [26], healthy diets [27], and lower stress levels [28], all of which contribute to better vascular health and lower CVD risk. While centenarians are not immune to the effects of vascular ageing, they seem to avoid the most severe vascular deterioration, which may be a key factor in their exceptional longevity. To gain a deeper understanding of the molecular mechanisms underlying this phenomenon, more studies on vascular ageing in centenarians are needed. However, conducting such research is challenging due to the fragility of centenarians and the ethical considerations involved.

## Intrinsic and extrinsic factors associated with vascular health in centenarians

**Fig. 2:**
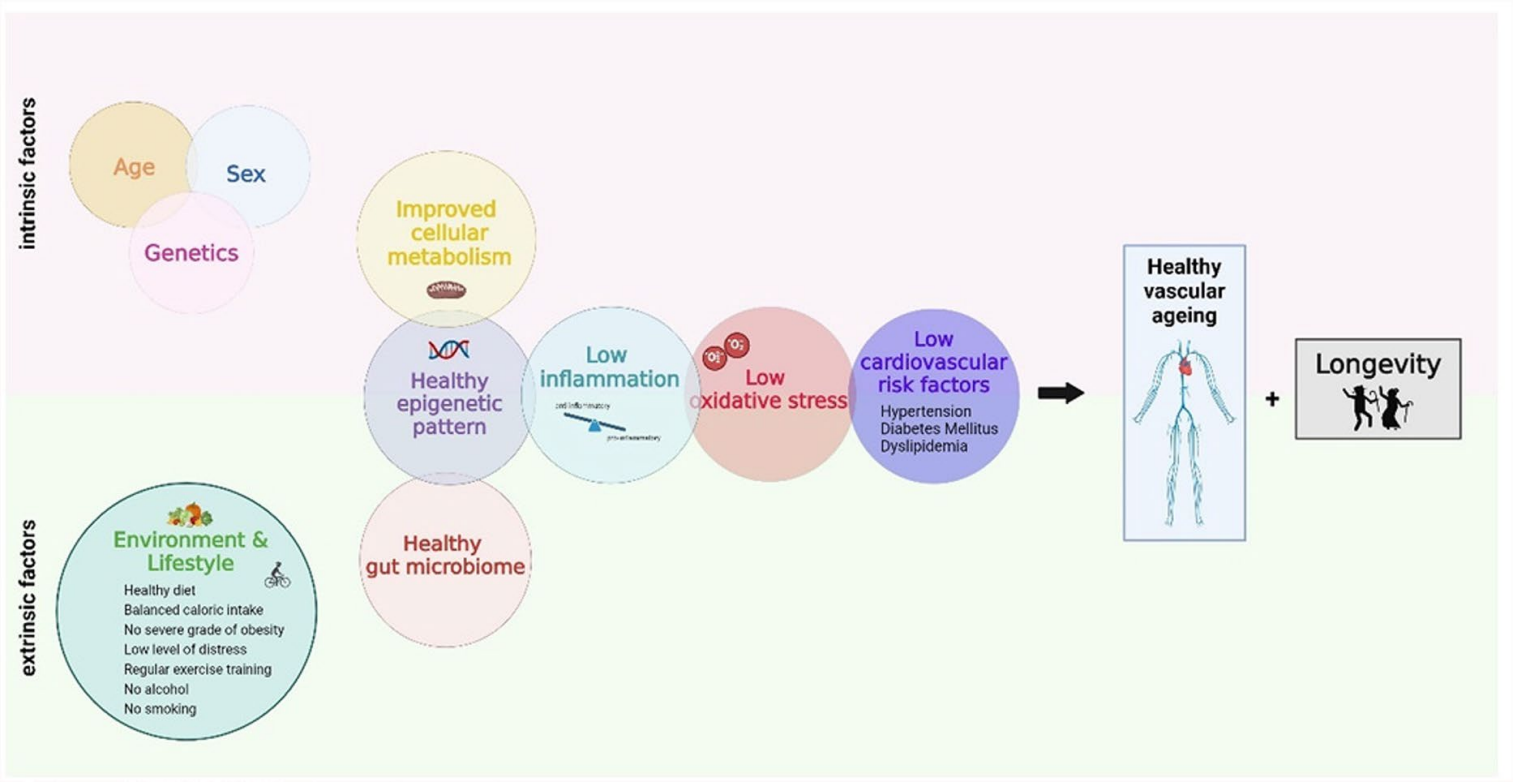
Factors affecting healthy vascular ageing and longevity. The illustration schematically depicts factors that contribute to healthy vascular ageing and could be key to longevity in centenarians. The factors are categorized into intrinsic factors (age, sex, and genetics) and extrinsic factors (environmental and lifestyle). The figure was created with BioRender.com

Centenarians show special genetic and non-genetic characteristics potentially contributing to their extreme longevity. In the context of CVD, the factors underlying their resilience against age-related vascular deterioration offer valuable insights into healthy ageing and are classified here into two main groups, intrinsic (non-modifiable) and extrinsic (modifiable) factors (Fig. 2). The genetic signature and sex are two of the main representatives of intrinsic factors influencing the vasculature. Extrinsic factors, such as lifestyle and environmental factors, may also exert a significant influence [29]. Centenarians often exhibit habits conductive to vascular health, such as a balanced diet rich in antioxidants and omega-3 fatty acids, regular physical activity contributing to improved endothelial function, and effective stress coping mechanisms reducing cortisol levels and inflammatory responses. Socioeconomic factors like access to healthcare and social support also play a pivotal role here.

### Intrinsic factors

Women outnumber men not only in terms of average life expectancy (84 years for women versus 79 years for men) but there are also twice as many women < 85 years than men in Europe [30]. Numerous factors contribute to this disparity, including biological, social, and behavioral differences, which will be discussed in more detail later in this review. In general, women show a lower susceptibility to CVD and a more robust immune system [31, 32]. The female sex hormone estrogen has been proposed to confer protective effects against heart disease and osteoporosis, but the more prominent effect might come from the healthier lifestyle habits. Moreover, social aspects including stronger social networks and greater willingness to seek medical care may also play a key role in the longer life expectancy of women [33]. Consistently, men are more prone to signs of vascular ageing than women [34]; however, sex differences become less pronounced when analyzing older adults [34, 35]. This could be at least partly linked to the incidence of menopause in mid-aged women [36, 37]. Remarkably, the age-related increase in carotid Intima-Media-Thickness (IMT) and adventitial diameter are significantly enhanced during post-menopause (Table 1) [34, 38]. Conceivably, menopause is considered to be a risk factor for CVD incident in women [39].

Accumulating evidence supports the genetic basis of longevity and reduced prevalence of morbidities in centenarians [40, 41]. Particularly, the prevalence of CVD is reduced in the offspring of centenarians [2]. In this context, genetic polymorphisms are crucial contributors to interindividual variations and can significantly influence the trajectory of healthy ageing. Certain genetic variants have been associated with a higher likelihood of reaching an advanced age while maintaining good health, including genes involved in cellular repair mechanisms, oxidative stress response, and inflammation [24]. Variations in genes encoding proteins involved in endothelial function, such as nitric oxide synthase (eNOS) and endothelin receptors, could impact blood vessel health and contribute to longevity [25, 42]. Genetic polymorphisms associated with antioxidant enzymes, such as superoxide dismutase and glutathione peroxidase, can mitigate oxidative stress and protect against age-related vascular damage. Moreover, variants in genes related to lipid metabolism, such as those encoding lipoprotein receptors and apolipoproteins, could influence cholesterol levels and reduce the risk of atherosclerosis and vascular disease [43].

Variations in genes involved in the regulation of inflammatory pathways, such as interleukins and tumor necrosis factor (TNF), are associated with attenuating age-related inflammation and preserving vascular integrity [44]. Inflammatory genes influence the development and progression of age-related vascular disorders such as atherosclerosis and hypertension. Various genetic polymorphisms within these genes have been identified as key contributors to the inflammatory response associated with healthy ageing and longevity [45–49]. This includes genetic variations in genes for angiotensin converting enzyme (ACE), apolipoprotein E (APOE), forkhead box protein 3A (FOXO3A), transmembrane glycoprotein KLOTHO, the klotho haplotype KL-VS, and IL-6 [50]. Genetic variations leading to decreased cytokine production are associated with a lower likelihood of experiencing cardiovascular complications [51, 52], whereas elevated pro-inflammatory cytokine levels are linked to pathological processes, as observed in atherosclerosis [53, 54]. A genetic association with reduced interferon (IFN)-γ production markedly enhances the likelihood of attaining prolonged longevity [47]. Furthermore, reports indicate that decreased inflammation and lower levels of reactive oxygen species (ROS) in individuals with extended lifespans are attributed to elevated levels of antioxidants like glutathione [55]. In this case, the positive effect on ageing might be based on gene polymorphisms that regulate inflammatory mediators and oxidative stress pathways. APOE4 has been shown to modulate ROS levels in endothelial cells [56] and FOXO3A regulates reactive oxygen metabolism by inhibiting mitochondrial gene expression [57, 58], while KLOTHO and IL-6 may exert their function by modulating cellular ROS levels [59].

Alongside a lower frequency of inflammatory diseases in comparison to other older people (< 100 years old), centenarians show increased antimicrobial, anti-inflammatory agents, and antioxidants as well as higher reductase superoxide dismutase activity levels compared with other aged groups, which might contribute to their longevity [55].

Like other ageing individuals, centenarians undergo inflammaging, a chronic low-grade inflammation, but they display a unique ability to counteract its effects. Centenarians tend to have lower levels of inflammatory markers typically increased in the general elderly population. They exhibit a more favorable balance between pro-inflammatory and anti-inflammatory responses, with higher levels of protective molecules like IL-10, which aids in the regulation and mitigation of chronic inflammation [19, 20]. In addition, this age group exhibits a shift to higher concentrations of IL-4 and lower levels of IL-2 and interferon (IFN)-γ, which points to a switch towards an anti-inflammatory profile [60]. Even neutrophil function [61], which is diminished in normal aged individuals [62–65], stays well preserved in centenarians [66]. Zhou et al. suggest that centenarians alleviate the effects of inflammaging by decreasing the ratio of Th17 and regulatory T-cells and forcing them into an anti-inflammatory secretory phenotype [19]. An elevated number of regulatory T-cells (Tregs) may protect against atherosclerosis. By secreting anti-inflammatory cytokines, Tregs can limit the activity of pro-inflammatory cells within vascular tissues, thereby reducing the formation and progression of atherosclerotic plaques. In centenarians, the higher presence of Tregs likely mitigates the effects of chronic inflammation, lowering the risk of atherosclerosis despite advanced age [19, 67]. An increase in cytotoxic activity in centenarians may be an important adaptation in the later stages of ageing, when the immune system more frequently needs to eliminate abnormal or infected cells. A recent study revealed an increase in cytotoxic CD4 T-cells in supercentenarians (reached 110 years) using single cell transcriptomics [68]. This T-cell population is proposed to be involved in antiviral immunity [69] and tumor control [70] but is also implicated in the development of autoimmune diseases. Interestingly, the prevalence of autoimmune diseases in centenarians is significantly lower compared to younger age group [71]. In general, hyperstimulation of the immune system will lead to the production of autoantibodies and auto-reactive T- and B-cells [72]. Centenarians may either be able to overcome immune hyperstimulation or may not have been exposed to strong external stimuli. Additionally, their genetic and epigenetic backgrounds could offer protection against hyperstimulation [73].

### Extrinsic factors

#### Lifestyle factors

Dietary patterns are a key group of modifiable lifestyle factors that influence successful ageing [27, 29, 74–78]. Current evidence supports the notion that engaging healthy behaviors, including healthy dietary habits, are differential features in centenarians [74–77, 79–87].

Adherence to healthy dietary habits, along with other lifestyle factors, is widely recognized as key factors in preventing CVD [88, 89]. Its association with lower mortality rates, both from cardiovascular causes and all-cause mortality, has been documented in previous epidemiological studies [90, 91]. A recent systematic review highlighted the use of various diet quality scores to assess their predictive role as risk factors for CVD, incident CVD, and CVD-related mortality across different populations [92]. However, few studies have specifically investigated the relationship between dietary quality scores and CVD protection in centenarians [74, 83, 85].

While countries with higher ratios of centenarians differ in lifestyle and culture, there seems to be common factors that contributed to the increased number of centenarians in these countries. Therefore, identifying these key factors linked to longer life spans and understanding their physiological and molecular mechanisms might pave the way to providing valuable insights that can be applied globally.

#### Caloric restriction

Consuming fewer calories without malnutrition (caloric restriction) may play a key role in the extended lifespan of centenarians. Although its beneficial effects on lifespan in humans are less clear [93, 94], this regimen may improve vascular health, encompassing enhanced endothelial function, reduced oxidative stress, and low-grade inflammation, thus, contributing to reduced incidence of age-related disorders. In the CALERIE (Comprehensive Assessment of Long-term Effects of Reducing Intake of Energy) study, 218 participants (66 men, 152 women, 21–50 years) received 25% less calories over two years. The participants showed a significant reduction in atherosclerotic risk factors apolipoprotein B (ApoB) and glycoprotein acetylation (GlycA) as well as a reduction in systolic, diastolic blood pressure, and total cholesterol levels relative to ad libitum control groups [95].

Many studies have reported that centenarians have similar lifestyles, including daily physical activities, moderate alcohol consumption, spiritual beliefs, and strong family and neighborhood ties, even though the centenarians are geographically and culturally different from each other. Additionally, there is a similarity in the physiological parameters of centenarians when compared to young volunteers (ages between 21 and 50 years), who participated in a 10–30% caloric restriction regimen from 10 weeks to up to two years [95, 96]. There is a decrease in systolic and diastolic blood pressure, glucose, insulin, and leptin levels, while an increase in insulin sensitivity and adiponectin levels has been observed in both centenarians and caloric restricted cohorts [97, 98].

Caloric restriction requires regulatory proteins that could sense food deprivation, thereby leading to a physiological response characterized by an improved mitochondrial homeostasis. Nicotinamide adenine dinucleotide (NAD)^+^-dependent protein deacetylases, particularly sirtuin 1 (SIRT1), is one of this sensor proteins that links cell energetics and lifespan [99]. Moreover, the relative abundance of tissue uncoupling proteins (UCPs) is to increase during caloric restriction in several tissues, which leads to reduced ROS production. Among their representatives, UCP2 is ubiquitous; therefore, it has been proposed as a key driver in ameliorating oxidative stress and macrophage immunity, and thus decreasing ageing progression in different tissues [100]. Over the last two decades, accumulating evidence showed that caloric restriction has potential to therapeutically modulate ageing [101, 102]. In this regard, the use of caloric restriction mimetics, such as resveratrol, a SIRT1 enhancer, and metformin, a drug administered to diabetic patients, has shown promising results on preventing ageing processes [103, 104] and to some extent exert favorable effects in the vasculature by ameliorating mitochondrial dysfunction [105–107]. The activation of an anti-oxidative response is thought to be related to decreased levels of 9-HODE and 9-oxoODE, which are markers of lipid peroxidation, and oxidative products of linoleic acid, and increased concentration of 8,9-EpETrE, a major cytochrome P450 metabolite. Additionally, the same cohort showed a decrease in serum tryptophan concentration, associated with ageing and age-related inflammation. Besides, centenarians showed higher levels of 15-hydroxy-eicosatetraenoic acid (15-HETE), which has anti-inflammatory properties [58]. While further research is needed to fully understand the effects of caloric restriction on human longevity, current evidence suggests that adopting a calorie-restricted diet may be beneficial for promoting healthy ageing, preserving vascular health, and extending lifespan. This is exemplified by the remarkable longevity observed in centenarians who adhere to such dietary practices.

#### Physical activity

Increased physical activity and especially involvement in regular exercise (such as brisk walking) are the main life-style behaviors that contribute not only to longevity but also to increased health span. Indeed, a cross-sectional epidemiological study (involving 1,907 centenarians in Japan) showed that involvement in regular exercise was associated with health and autonomy/independence level of centenarians [108]. Additionally, Johansson and Thorvaldsson examined identical and same-sex fraternal twin pairs, showing that longer survival after 80 years was associated with greater muscle strength, better lung function, vision (but not hearing), and cognitive function (self-evaluated and measured) [109]. Exercise exerts its beneficial effects by reducing the incidence of chronic disease [110] and especially by delaying ageing-induced cardiovascular impairment. Specifically, regular physical activity and exercise training could delay declines in fitness levels and mitigate many cellular and systemic processes associated with ageing by the following: (i) preserving mitochondrial health in skeletal muscle through the repeated activation of peroxisome proliferator-activated receptor-c coactivator-1a (PGC-1a), which induces mitochondrial biogenesis [111, 112], (ii) decreasing chronic oxidative stress and preventing inflammation, (iii) improving cardiovascular health, and (iv) counterbalancing the age-related deterioration of pulmonary function observed in centenarians by enhancing the mechanical efficiency of skeletal muscles [113]. Another important benefit of exercise is the delay of the age-associated decline in muscle mass and strength, and prevention of sarcopenia, frequently observed after the 8th decade of life [114, 115]. This is achieved by regulating the balance between anabolic activities driven by insulin-like growth factor-1 (IGF-1) and catabolic activities mediated by factors such as proteasome Atrogin-1 and autophagy–lysosome MuRF-1. Additionally, regular physical activity improves energy supply through mitochondrial ATP production [116, 117].

In the vascular system, regular exercise can improve peripheral vascular function [118] and arterial compliance [119, 120] as well as skeletal muscle capillary content [121], resulting in improved tissue oxygen and nutrient delivery. Together, these improve physical fitness/function (as assessed by peak oxygen consumption, VO_2_ peak), due to maintained or even improved muscle perfusion and O_2_ delivery to skeletal muscle [122], and contribute to lower the cardiovascular risk [123, 124]. The improved capillarization in the skeletal muscle of habitually active older adults can be attributable to regular elevations in shear stress [125] and circulating angiogenic factors, such as vascular endothelial growth factor (VEGF) [126], both of which stimulate endothelial cell proliferation, resulting in an increased capillary density. Interestingly, exercise-induced angiogenic responses appear to be independent of age, with older adults exhibiting similar improvements in skeletal muscle capillary density compared to younger individuals [127]. The acute exercise-induced shear stress improves various properties of vascular endothelial function. The elevated blood flow during exercise increases shear and promotes release of endothelial-derived nitric oxide (NO), resulting in vasodilation, and allowing better tissue perfusion, and, thus, better oxygen and nutrient delivery [128, 129]. The greater shear stress also activates intracellular signaling pathways that prevent endothelial dysfunction and atherosclerotic plaque formation, induce beneficial vascular remodeling, and promote angiogenesis. Therefore, exercise could reduce the risk of endothelial dysfunction-associated diseases in older people, such as hypertension and CVD [130]. However, studies involved in determining the relationship between lifelong physical activity and vascular parameters in this group of very old individuals are lacking.

To summarize, being physically active, along with a healthy lifestyle, is one of the most important things to keep the cardiovascular system healthy and contribute to the longevity of older individuals and may contribute to the exceptional longevity observed in centenarians.

#### The gut microbiome

The gut microbiome composition may have a role in the prevention of age-related diseases by influencing systemic immune function and resistance to infections [131]. The composition of gut microbiota of centenarians differs from that of non-centenarians [132, 133]. Remarkably, daily dietary patterns profoundly influence the composition of the gut microbiome in humans [134]. This suggests that the beneficial effects of dietary adaptations associated with enhanced longevity may be, at least in part, attributable to favorable changes in gut microbiota composition. Emerging evidence suggests that alterations in the composition and function of the gut microbiome may contribute to age-related vascular dysfunction and CVD [135, 136]. The gut microbiota plays a crucial role in regulating host metabolism, immune function, and inflammation, all of which are implicated in vascular ageing processes [137–140]. Normal ageing reduces the diversity of the gut microbiota, while it remains biodiverse and enriched in *Akkermansia*, *Bifidobacterium*, and *Christensenellaceae* in centenarians [58, 135, 141]. The biodiversity of the microbiota promotes anti-inflammatory conditions that potentially contribute to metabolic homeostasis and diminish low-grade inflammation that is caused by obesity or cardiometabolic disease [58, 142]. These centenarian-specific microbiota profiles are thought to contribute to their remarkable health span and resilience to age-related diseases. Centenarians exhibit elevated metabolic activity in glycolysis and the fermentation of short-chain fatty acids and a diminished carbohydrate turnover [143]. Besides, findings of a specific bile acid composition in centenarians may explain their reduced risk of pathogenic infection [144]. The resulting stable equilibrium in gut microbiome composition can limit abnormal vascular inflammation and enhance vascular health. Along with the bacterial microbiome, recent studies also support the contribution of age-dependent patterns of gut virome diversity to healthy lifespan in centenarians [145]. However, further research is still required to assess the relationship of age-specific signatures with cardiovascular health in centenarians.

#### Use of medicines

There are few research studies on drug use in centenarians, with data often obtained from collections with a small number of participants and selected samples without comparisons with other age groups [146–149]. Although the use of medications is generally thought higher across extreme aging, several studies indicate that the use of medications is generally lower in centenarians compared with younger groups of age [150–152]. However, it may depend on the class of drugs being considered. For instance, a recent study using nationwide register data revealed that the use of analgesics, hypnotics/sedatives, and anxiolytics was higher in centenarians; instead, the group of antidepressants was lower than nonagenarians and octogenarians [153].

Relative to cardiovascular diseases, and according to the previous study, the centenarians used less antithrombotic agents, beta-blockers, and angiotensin-converting enzyme (ACE)-inhibitors than octogenarians and nonagenarians [153], being this finding consistent with the common notion that cardiovascular drug therapy is prescribed to a lesser extent in centenarians compared with younger groups of age [147, 154]. The reason underlying such differences is unknown, but could be, at least partly, attributed to the adherence to healthier lifestyle patterns by centenarian survivors compared with other younger age groups.

#### Epigenetic modifications as habit/environmental sensors

Lifestyle conditions and environmental agents may lead to epigenetic DNA/protein modifications that regulate transcriptomics [155]. Several studies have identified specific epigenetic changes including DNA methylation changes, histone modifications and differentially expressed microRNA (miRNA) in ageing that may contribute to exceptional longevity of centenarians [156–158]. Interestingly, the expression profile of centenarians was similar to that of young individuals but differed significantly from individuals over 80 years. By analyzing the non-coding RNA profile, seven non-coding RNAs were found upregulated in centenarians, four of known function (SCARNA17, miR-21, miR-130a, and miR-494) and two of unknown function (miR-1975 and miR-1979) [156]. Findings suggest that achieving healthy ageing in centenarians and supercentenarians relies not only on preserving younger epigenetic profiles, but also on the presence of specific epigenetic regions [159]. In a parallel manner compared to the methylome of newborns, the methylome of centenarians shows attenuated levels of DNA methylation alongside a diminished pairwise correlation in the methylation status of adjacent CpG sites [160]. Moreover, histone modifications actively regulate the expression of age-related genes, shaping cellular responses to ageing processes, promoting longer life and healthy ageing by modulating gene expression, and maintaining the epigenetic stability. Across various organisms, research has also highlighted varying expression patterns of miRNAs which regulate the key gene expression by ageing [161, 162].

The exposure to dietary patterns [163] and hypocaloric regimens [164] could shape the epigenome. In this context, changes in the diet could cause a shift in the epigenome based on age of a person [164]. Life-long caloric restriction, starting from 14 weeks of age until 24 months, was shown to prevent age-related differential methylation levels. Moreover, caloric restriction in mice has been reported to result in methylation profiles similar to those of younger mice in the hippocampal region of the brain [165].

Overall, further studies are needed to comprehensively explore the relationship between age-specific molecular signatures of centenarians and their functional consequences, particularly those pertaining to epigenetic modifications.

#### Infectious diseases

Centenarians often exhibit an immune profile that is better adapted to managing low-grade inflammation while still maintaining the ability to combat infections. Other aged individuals show an increased susceptibility to infections, mainly because of the decreased efficacy of adaptive immunity to contain microorganisms [166].

AMPs may help by directly neutralizing pathogens and preventing infections that could otherwise accelerate aging or trigger systemic inflammation. Studies suggest that this resilience is partly due to their unique immunological profile [167] and a preservation of adaptive immunity [166, 168]. Additionally, their gut microbiota often harbors a greater abundance of beneficial microbes, which may enhance immune response and reduce infection risk [145].

Antimicrobial peptides (AMPs) likely contribute to this protection as well by directly neutralizing pathogens and regulating immune responses [169]. AMPs are small peptide molecules of less than 50 amino acids encoded by individual genes [170]. Certain AMPs, such as human cathelicidin LL-37 and defensins, are associated with healthy ageing. Dysregulation of these peptides is linked to infections and chronic diseases [171, 172]. In conclusion, AMPs are promising candidates not only to understand the mechanisms of longevity, but also as targets for interventions for age-related diseases.

To summarize, centenarians show a combination of genetic resilience, well-regulated immune system, healthy microbiome, and beneficial lifestyle habits that contribute to their resistance to infections.

## Conclusion and future perspectives

Longevity is a complex process influenced by both genetic and non-genetic factors. The prevalence of individuals [166] aged 95 and older, known as centenarians, has been increasing in various regions worldwide. This specific group displays lower incident rates of all-cause mortality, including cardiovascular-related deaths. Compelling epidemiological data strongly suggest that centenarians maintain a favorable balance of advantageous factors (e.g., a healthy diet and regular physical activity, with no alcohol consumption nor smoking) alongside disadvantageous factors (e.g., environmental stressors or genetic predisposition to complex cardiometabolic diseases and cancer) throughout their life.

Centenarians frequently display improved [i] glucose management and insulin signaling, as well as [ii] a more favorable plasma lipid and lipoprotein profile. They also show fewer overall signs of oxidative stress compared to aged controls [173, 174]. While a significant proportion of centenarians with diabetes also have hypertension, they tend to experience fewer diabetes-related complications, such as lower prevalence rates of peripheral arterial disease, neuropathy, and congestive heart failure [175]. Additionally, this population maintains relatively high cognitive function and physical fitness and is highly resistant to diseases, such as stroke, metabolic syndrome, and CVDs. This suggests that various factors may play a role in preventing or mitigating these adverse outcomes among centenarians. Here, it seems that the combination of a reduced production of pro-inflammatory cytokines regulated by polymorphisms in specific genes and an increase in antioxidants has a positive effect on their health status. The delay in age-induced vascular impairments observed in centenarians has been linked to improved lifestyle conditions, with healthy dietary patterns and regular physical activity. Given that dietary patterns profoundly influence the composition of gut microbiota, it can be postulated that these changes may also, at least in part, help attenuate low-grade inflammation, thereby reducing the subsequent risk of CVDs.

Genetic, environmental and lifestyle factors are associated with changes in the functional and structural properties of the vascular wall leading to premature ageing. Clinical evidence indicates that mortality linked to cardiometabolic disorders increases with age and is higher in individuals suffering from age-related diseases. Biomarkers of vascular ageing that have been linked to high risk of developing accelerated vascular damage [17, 34] could be used as therapeutical targets to improve the outcome of patients with age-related cardiovascular diseases. Beyond the potential involvement of telomere’s length, an essential but unspecific feature of ageing [176] and a range of candidates as vascular ageing biomarkers have been proposed, including arterial stiffness, blood pressure, endothelial dysfunction, and carotid IMT [17] (Table 1). However, it should be noted that gender- and sex-specific data are lacking in the literature due to the limited population size of centenarians. Therefore, studies involving this population should be well-designed, with a focus on specific specimen storage, data collection, and sharing platforms. This approach will facilitate easy access to biological samples from centenarians, enabling more efficient research on topics such as vascular ageing.

## Data Availability

This review did not generate any new data. All data used in this study are available from the referenced sources.
